# A systematic review of impacts of COVID-19 on depression and anxiety among general populations around the world

**DOI:** 10.3389/fpubh.2025.1659671

**Published:** 2025-10-23

**Authors:** Jianbo Hou, Wenjie Duan, Yuqian Wang, Yujing Liao, He Bu, Wenlong Mu, Xiaoqing Tang, Dong Liu

**Affiliations:** ^1^School of International Education, Xi'an Technological University, Xi'an, China; ^2^Social and Public Administration School, East China University of Science and Technology, Shanghai, China; ^3^School of Journalism and Communication, Wuhan University, Wuhan, China; ^4^College for Public Wellbeing, Shanghai Open University, Shanghai, China; ^5^Department of Social Work, Shanghai Business School, Shanghai, China

**Keywords:** mental health, anxiety, depression, systematic review, COVID-19

## Abstract

**Background:**

The COVID-19 pandemic has profoundly impacted global mental health, with significant disparities in depression and anxiety observed across populations and countries. Existing literature highlights the role of social determinants of health (SDH) in shaping mental health outcomes, yet systematic reviews synthesizing these impacts across diverse socioeconomic and policy contexts remain limited. This study provides an overview of how COVID-19 is affecting depression and anxiety among general populations, alongside inequalities driven by the SDH.

**Methods:**

Six databases (CNKI, Embase, PubMed, Scopus, Cochrane Library, Web of Science) were searched from March 2020 to February 2024. Inclusion criteria encompassed cross-sectional/longitudinal studies assessing depression/anxiety in adults (≥18 years) using validated scales (e.g., PHQ-9, GAD-7). After screening 4,916 records, 59 studies met eligibility criteria. Quality assessment utilized the Joanna Briggs Institute tool, and data extraction covered study characteristics, outcomes, and SDH factors. This review is registered with PROSPERO: CRD420251023201.

**Results:**

Among 59 studies (39 from low- and middle-income countries [LMICs]; 16 from high-income countries [HICs]), younger individuals, women, and socioeconomically disadvantaged groups exhibited heightened vulnerability to depression and anxiety. High-income countries with stringent lockdowns (e.g., the U.S., France) reported sustained psychological distress, while nations adopting effective early containment strategies saw mental health improvements over time. Population-level determinants, including healthcare infrastructure and policy stringency, significantly influenced outcomes. Low-resource settings faced worsened mental health burdens due to prolonged restrictions and limited medical access. Individual and community-level factors such as unemployment, housing instability, and low social support amplified risks. Temporal trends revealed worsening mental health during extended lockdowns and disparities in recovery trajectories across regions.

**Conclusion:**

The COVID-19 pandemic exacerbated mental health inequalities, disproportionately affecting specific groups and underscoring the interplay of SDH. Tailored interventions addressing socioeconomic vulnerabilities, enhancing social support, and balancing infection control with psychological well-being are critical.

**Systematic review registration:**

https://www.crd.york.ac.uk/PROSPERO/view/CRD420251023201, identifier CRD420251023201

## 1 Introduction

Public health emergencies, defining as much by their triggering events as by their health consequences (1), profoundly impact social systems, policies, and health. The health consequences of public health emergencies possess the potential to exceed the routine capabilities of the community, characterized by a scale, timing, or unpredictability that poses a significant threat to the existing response capacity (2). With the raise of interaction between nature and human actions, emerging viral zoonoses are a critical threat to public health (3). Historical analysis reveals several paradigmatic examples of such emergencies. The 1918–1919 H1N1 influenza pandemic resulted in the death of approximately 50 million people worldwide, accounting for 3–5% of the global population at that time. It tremendous pressure on public health systems, medical resources, and social life (4). Another prominent example emerged in 2002–2003 with the global outbreak of severe acute respiratory syndrome (SARS), which resulted in 8,098 confirmed infections and 774 deaths, yielding a case fatality rate of 9.60% (5). In December 2019, COVID-19 caused a global pandemic that resulted in more than 6.5 million deaths (6). The World Health Organization (WHO) declared the COVID-19 outbreak an international public health emergency on January 30, 2020 (7). As the most significant public health event in recent years, it has profoundly impacted mental health (8).

The COVID-19 pandemic has triggered a wide range of mental health symptoms across different populations, with significant disparities observed globally. Large-scale international studies have begun to systematically assess these impacts. An international study administered in 30 countries across the globe assessing the health-related impacts of the COVID-19 pandemic on individuals’ fear of the pandemic examined its global effects on lifestyle behaviors, fear, depression, and perceived community needs (9). A cross-sectional study focused on fear in general populations on perceived fear of common diseases, life events, and environmental threats in 30 countries (10). Furthermore, the mental health disparities among the general public caused by COVID-19 have been documented by many scholars. A network analysis of a large international observational study figured that, quality of life, emotional distress, and the overall amount of exercise are key intervention components for improving overall lifestyle, overall health and overall health via lifestyle in the general population, respectively (11). Comparative studies reveal that mental health burdens varied significantly across countries, often influenced by economic and epidemic severity factors. Li et al. (12) assessed and made cross-country and cross-region comparisons of the global impacts of COVID-19 and preparation preferences of pandemic. Countries with a high-income level or medium to high COVID-19 severity reported higher perceived mental burden and emotional distress. In Slovenia, the risk of depression, anxiety, and stress was well controlled during the first wave of COVID-19 when the daily record of positive cases was only 61 and the healthcare system was not overwhelmed, but it was not well controlled during the second wave (the epidemic was again announced on 18 October 2020) when infections spread rapidly and exponentially, strict measures were prolonged, and Slovenia became one of the hardest-hit countries (13). In Brazil, where the pandemic emerged amid political and scientific conflict, a survey conducted from May 22 to June 5, 2020, 5 months after the first confirmed case, found a high prevalence of depression (46.4%), anxiety (39.7%), and stress (42.2%) (14). Around the same period, in Malaysia, during the conditional movement control order (CMCO) from 13 May to 9 June 2020 followed by a more lenient recovery movement control order (RMCO) from 10 June to 31 August and extended until 31 December, there were increased depressive, anxiety, and stress symptoms from 12 May to 5 September 2020, with depression showing the greatest rise (15). Overall, these findings highlight that the prevalence and trajectory of depression, anxiety, and stress during the COVID-19 pandemic varied across countries, reflecting differences in pandemic severity, public health measures, and socio-political contexts.

It is evident that, there are significant differences in the prevalence of depression and anxiety among different countries, and the prevalence of depression and anxiety during the same period are also different. These differences are not only attributable to the timing and severity of the outbreak but are also influenced by factors such as age, gender, occupation, lockdown policies, race, and other demographic variables.

A substantial number of social theories pertaining to health have been employed to elucidate the phenomenon of variability in the public’s mental health that have emerged as a consequence of the COVID-19 pandemic. Some scholars explore this issue from the perspective of social stratification theories. Their studies reported that the scale of a society’s income inequality is a determinant of population health (16). Social factors such as socioeconomic status and social support are likely posited as “fundamental causes” of disease. These factors, by virtue of their embodiment of access to critical resources, exert influence on multiple disease outcomes through diverse mechanisms (17). The structural determinism view is that, systemic power structures such as racism and sexism indirectly harm the health of vulnerable groups through institutional exclusion (18). For people of color, the systemic and structural racism produce, condone, and perpetuate widespread unfair treatment and oppression of them, with adverse health consequences (19). Social capital theory hold the view that differences in trust, reciprocity, and resource flow in social networks such as strong or weak community cohesion affect the health level of the group (20).

Given the multifaceted ways such as those rooted in socioeconomic status and systemic power structures, influence health outcomes, it is imperative to adopt a more comprehensive perspective to understand and address the public mental health exacerbated by the COVID-19 pandemic (18–20). The model of social determinants of health (SDH) was first presented by the scholars Dahlgren and Whitehead in 1991 (21). In 2008, the WHO Commission on Social Determinants of Health further elaborated the definition in its report Closing the Gap in a Generation. WHO defines SDH as “the conditions in which people are born, grow, live, work and age,” conditions or circumstances that are shaped by families and communities and by the distribution of money, power, and resources at global, national, and local levels and affected by policy choices at each of these levels (22). Individual and group-level social determinants include gender, race, social class, education, income, occupation, employment status, housing tenure, immigration status, disability status, and social capital. Population-level determinants encompass health service provision, access to essential services, medically underserved or health professional shortage areas, and public expenditures on safety, social, and welfare services (23).

To date of research, there has been a lack of systematic reviews that summarized the impact and manifestations of depressive and anxiety symptoms among the general population during disease outbreaks, particularly exemplified by COVID-19, across countries with varying levels of development. To better understand the social structural roots of mental health inequalities across countries with different levels of economic development, this study was framed within SDH. Focusing on three dimensions: individual social determinants, population-level determinants and community-level social support determinants, this research deepens the theoretical understanding of health inequalities, particularly revealing how social structures in health emergencies shape mental health differences, offering a new perspective for cross-national comparative studies in sociology and public health.

## 2 Method

### 2.1 Study design

The design of this review followed the Preferred Items for Systematic Reviews and Meta-Analyses (PRISMA) statement guideline (24). The protocol for this systematic review was prospectively registered on PROSPERO (registration number: CRD420251023201).

### 2.2 Search strategy

Following PRISMA procedures, China National Knowledge Infrastructure (CNKI), Embase, PubMed, Scopus, Cochrane Library and Web of Science were searched. A large number of high-quality papers has been published in these databases, and similar studies were retrieved from the different sources (25). The systematic search was conducted from March 11, 2020, to February 3, 2024, beginning with the declaration of the novel coronavirus pandemic by WHO (26). Adhering to the PICOS principle, we utilized a combination of subject terms and free words for our searches. The Chinese key words include but not limited to “新冠肺炎,” “焦虑,” “抑郁” and “剬众,” while the English search words are “COVID-19,” “depression,” “anxiety” and “General population,” and the detailed search terms and combination can be found in Supplementary Table 1.

### 2.3 Eligibility criteria

A study would be included if it fully met the following criteria: (a) type of study: cross-sectional or longitudinal study, consisting of a single collection during the pandemic (begin with March 11, 2020). (b) study subjects: the general adult public (≥18 years old). (c) study variable: depression or anxiety. (d) outcome indicators: psychometric scales such as Generalized Anxiety Disorder Scale (GAD)-7, GAD-2, Patient Health Questionnaire (PHQ)-2, PHQ-4, PHQ-9, and Depression Anxiety Stress Scales (DASS)-21 were used during the data collection period. (e) published peer-reviewed Chinese and English journal literature, available in full text or by contacting the author to obtain relevant data.

A study would be likewise excluded if it met one of the following criteria: (a) qualitative study, case–control study or intervention study. (b) conducted in specific subgroups (e.g., children, adolescents, and clinical sample) or in special settings (e.g., hospitals, military). (c) variables are depression or anxiety scores with no objective or self-reported measures were provided. (d) outcome measures data incomplete or not available by contacting the authors. (e) abstracts, conference papers, and reviews.

### 2.4 Study selection

**Figure 1:**
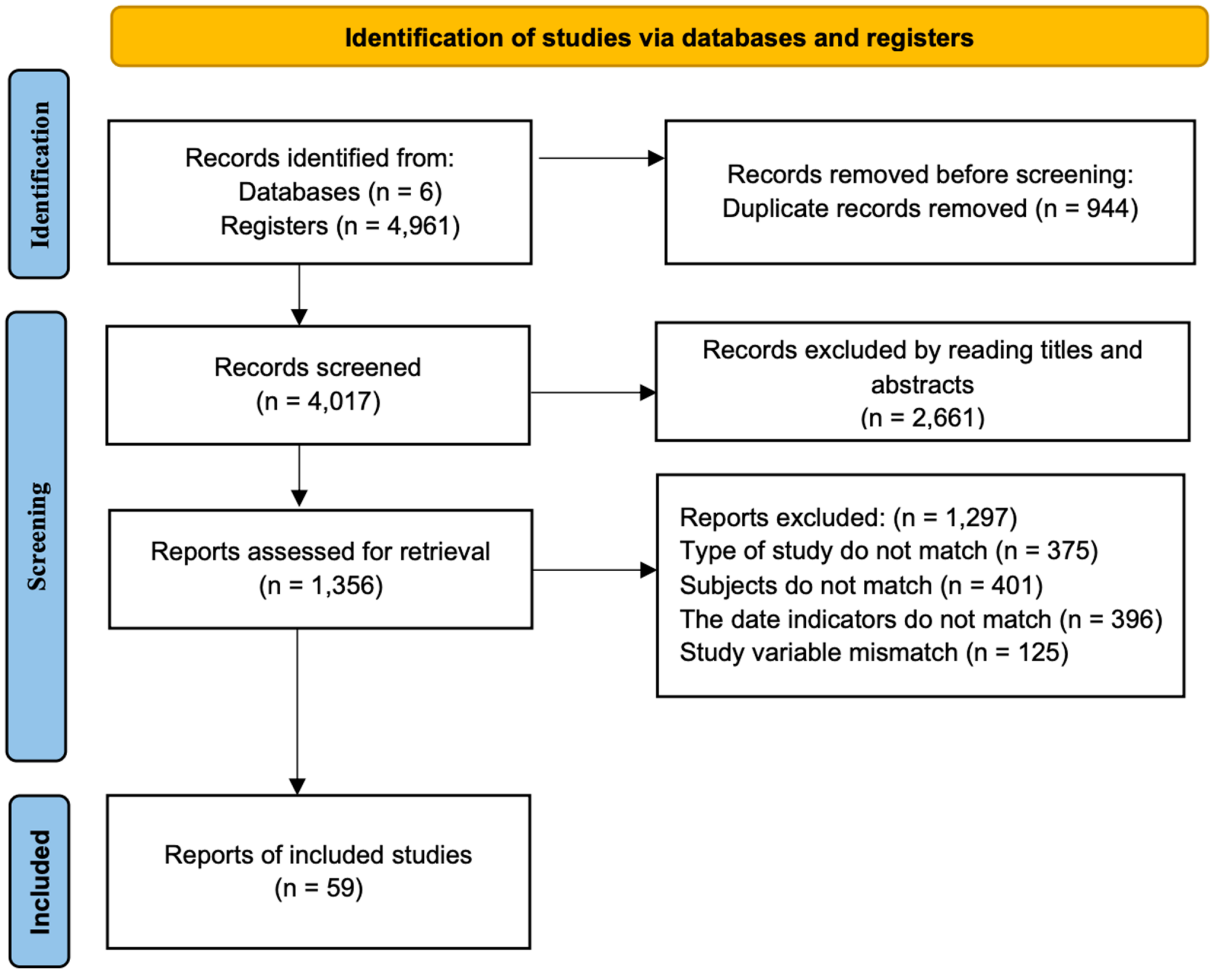
PRISMA flowchart of study selection.

Figure 1 showed the flowchart of screening. A total of 4,916 articles were initially identified. Preliminary screen resulted in the removing of 944 duplicates, and 2,661 articles with unqualified titles and abstracts. Among the remaining 1,356 articles, 1,297 were removed after reading the full-text articles according to the exclusion criteria above. In details, 375 articles were removed due to the type of study, 401 for unsuitable subject, 396 for mismatched the data indicators, 125 for discrepant study variables. Finally, there were 59 articles were included for analysis (see Supplementary Table 2 for details).

### 2.5 Quality assessment

The quality assessment conducted with the Joanna Briggs Institute’s critical appraisal tool, which is a particularly designed to assess studies of prevalence (27). The assessment consists with 9 items covering the article’s following 9 aspects: Was the sample frame appropriate to address the target population? Were study participants sampled in an appropriate way? Was the sample size adequate? Were the study objects and the setting described in detail? Was the data analysis conducted with sufficient coverage of the identified sample? Were valid methods used for the identification of the condition? Was the condition measured in a standard, reliable way for all participants? Was there appropriate statistical analysis? Was the response rate adequate, and if not, was the low response rate managed appropriately? A maximum of 9 points can be awarded for one study, with each item assessed as “YES” earning 1 point, while “NO,” “Unclear,” or “Not applicable” receive no marks. According to the scores, we classified the quality of articles as low level (with a score of 1–3), medium level (with a score of 4–6) and high level (with a score of 7–9).

### 2.6 Data extraction

The information we extracted from each study included source, countries, study aim, study types and main methods, sample size, measure tools, period of collecting and outcomes/key findings. If there were any doubts concerning the fulfillment of these criteria, it was resolved through discussions.

## 3 Result

### 3.1 The general results of included studies

A total of 59 studies were included in the analysis (refer to Supplementary Table 2), of which 39 focused on developing country and 16 focused on developed country and 4 of them surveyed both developing and developed countries.

Among them, 10 studies conducted separately in China, 5 in Iran, 4 in Italy, 3 in India, 3 in Malaysia, 2 in Brazil, 2 in Saudi Arabia, 2 in Korea, 2 in Sweden, 2 in Brazil, 2 in the United States, 2 in the United Arab Emirates (UAE) and 2 studies were conducted both in China and Iran, and China and Spain. One was conducted in both Czech and Slovakia and 1 study were conducted both in Italy and Isreal. Other countries or regions had 1 study each: Slovenia, Mexico, Bangladesh, Jordan, Greece, Libya, Japan, Hong Kong Special Administrative Region, China (Hong Kong SAR, China), French, Germany, Scotland, Australian, Serbia, Thailand, Poland, Canada and Belgium.

A total of 24 studies employed the Depression, Anxiety, and Stress Scale, while 35 studies utilized the PHQ. Among these 35, two studies used the PHQ-4, one study used the PHQ-12, and another one used the PHQ-15; all other studies utilized the PHQ-9. Similarly, for anxiety assessment, 27 studies used the GAD-7, whereas only one study employed the GAD-2. Notably, the most frequently used combination of measurement tools was the PHQ-9 and GAD-7, which were utilized together in 23 studies. Additionally, several other measurement instruments were also employed across different studies, including the Insomnia Severity Index (ISI)-7, the Quality of Life Scale −5 (QoL-5), the Impact of Event Scale-Revised (IES-R), the Subjective Well-Being Scale (SWB), the Center for Epidemiologic Studies Depresion Scale-7 (CESD-7), the Fear of COVID-19 Scale (FCV-19S), and the Acute Stress Disorder Scale (ASDS).

The data collection period for almost all studies was 2020 and 2021, and many studies had a segmented measurement time that span multiple stages, but only 4 studies involved data collection in 2022, and 1 study had a pre-measurement in 2018. The age range of study participants varied, although the participants in most studies belonged to the age group of 18 to 55 years. Certain studies with mixed participants were only partially included. Most of included studies are online studies or based on online survey. The review included 34 cross-sectional and 5 longitudinal studies, along with one case-controlled study, one prospective cohort study, one secondary analysis, one network analysis, and one propensity score–matched analysis.

### 3.2 Quality appraisal

Supplementary Table 3 displays the results of quality appraisal for the included 59 studies. Overall, 47 studies were rated as high-level quality and 12 studies were rated as medium-level, indicating a general good quality of studies. The included studies performed well in the aspects of scientific measurement tools, rigorous measurement process, and rationality of statistical methods, with no studies rated as “No.” The quality of population representation, recruitment and sampling needs improvement. Additionally, numerous research methods employed in the articles are, and the reliance on this result necessitates cautious interpretation when comparing differences between countries.

### 3.3 The trend of mental health over time

The phases of time had an undeniable impact on the mental health of people in various countries. A few studies elucidated unique mental health challenges faced during the early stages of the pandemic. Five months after the first case of contagion was registered on January 26, a survey conducted from May 22 to June 5, 2020, found that almost half of participants expressed symptoms of depression (46.4%), anxiety (39.7%), and stress (42.2%) (14). In contrast, in the U.S., during the early stages of the pandemic (April–May 2020), the average psychological impact was modest at the start and tended to decrease over time, with symptoms of stress, anxiety, and depression peaking at wave 1 (data collected April 20, or approximately 1 month into the US-based experience of the pandemic) (28). However, the study in Slovenia, conducted after the lockdown phase in July 2020, showed that the second wave was associated with a higher risk of depression, anxiety, and stress (13).

Longitudinal studies were commonly used (in Supplementary Table 2), focusing on the longitudinal development and changes in mental health conditions over time. A longitudinal analysis among the French population before and during the first and second COVID-19 lockdowns demonstrated significantly-declined mental health during extended lockdown periods, particularly under the more restrictive conditions of the second wave (29). The longitudinal studies in Greece and German documented a gradual deterioration in mental health over the pandemic, revealing the growing mental health burden over time, highlighted the role of cognitive and perceptual changes throughout the pandemic (30, 31). While Italy and Belgium gradually returned to pre-pandemic levels as restrictions lessened (32, 33). The general Chinese population improved their mental health from the early stages of the pandemic (T1: February 2020) to 8 months later (T2: October to December 2020), as pandemic control measures and public adaptation progressed (34).

### 3.4 The manifestation of mental health based on SDH

#### 3.4.1 Individual social determinants

##### 3.4.1.1 Age differences in anxiety and depression

Some of the demographic characteristics that may lead to higher rates of anxiety and depression. The most common factors are age and gender. Anxiety was significantly associated with female, being young and middle-aged (35). A total of 12 studies identified young age and female as the two most frequently reported factors linked to various COVID-related and psychosocial variables.

Several studies have reported that younger individuals are more vulnerable to outbreaks compared to older adults, with multiple explanations provided for this observation. Coping strategies during emergencies are age-dependent, significantly influencing mental health outcomes. A cross-sectional study assessing the mental health impact of COVID-19 across three age groups found that older adults were less likely than younger adults to employ problem-focused coping and exhibited lower levels of positive affect. Instead, they were more inclined to adopt adaptive and relaxed coping strategies (36). This difference in coping styles is often attributed to the greater social experience and resilience of older adults. Older adults’ resilience in response is stronger than youngers for having more stable social resources and more complex life experiences (37). Additionally, the psychological burden of crises has been shown to decrease with age (38). Furthermore, Beutel et al. (30) identified an association between younger age groups and lower economic status, as well as higher levels of loneliness Although loneliness levels remained similar to pre-pandemic baselines, younger individuals experienced a notable increase in loneliness during the pandemic.

Meanwhile, young people are more likely to suffer from unemployment and housing crises, largely due to the economic pressures associated with independent living. Michinaka et al. (37) identified several sociodemographic factors, including age, employment status, and fear or perceived risk of COVID-19 infection, as key contributors to mental health challenges. People experiencing homelessness (PEH) in younger age groups (18–34 years), with joblessness, heightened perceived infection risk, and elevated fear of COVID-19, were found to be at greater risk for depression and anxiety. A prospective cohort study conducted in Australia examined changes in mental health and help-seeking behaviors among young Australian adults during the COVID-19 pandemic. The study reported an increase in symptoms of depression and anxiety among this group. However, this rise was not matched by a corresponding increase in professional help-seeking. Many young adults engaged in self-help behaviors such as seeking social support (82.90%) and regular exercise (69.20%), both of which are associated with enhanced resilience. Nevertheless, they are lack of increased formal help-seeking, highlighting a gap in mental health service engagement for young age group (39).

##### 3.4.1.2 Gender differences in anxiety and depression rates

Regarding gender, females were more likely to exhibit trajectories characterized by greater vulnerability to symptoms of anxiety and depression (29). Women were particularly susceptible, especially women in the workplace or in the medical workplace. A cross-sectional study in Saudi Arabia revealed that male healthcare providers were less likely to experience anxiety (Beta = −0.22, *p* < 0.04), while nurses showed higher anxiety levels (Beta = 0.445, *p* < 0.026) (40).

Women do more easily to be affected for their passive roles in family relationships (41). This insecurity is reflected in significantly poorer sleep quality (42). Hubbard et al. (43) conducted a cross-sectional nationally representative survey via telephone in Scotland in June 2020 and found women exhibited poorer mental health. Moderating factors like loneliness, low social support, threat perception, and illness representations amplified the negative impacts on mental health for them.

Among the studies examining gender differences in mental health, four specifically addressed gender differences in depression and anxiety. Not all of the findings suggested worse mental health outcomes in females compared to males. Baseline data from the Omtanke 2020 Study in Sweden revealed variations in prevalence across sex, age, recruitment type, COVID-19 status, region, and seasonality. Notably, 43.4% of participants exhibited significant, clinically relevant symptoms in at least one of three mental health domains, with comorbidities being frequent; 7.30% had significant symptoms for all three outcomes (44). A survey conducted in Jordan reported higher stress scores in men (11.39 ± 0.469) than women (10.74 ± 0.33, *p* < 0.001), while women exhibited higher anxiety and depression scores compared to men (38). In the United States, a post-lockdown study during the COVID-19 pandemic found a higher prevalence of depression among males (45%) compared to females (32%), whereas the prevalence of anxiety was identical for both genders (42%) (45). Some studies have similarly reported that men exhibit higher levels of anxiety and depression compared to women (13, 46). Further investigation into specific symptoms is necessary to accurately assess the severity of mental illness and its variations between genders.

##### 3.4.1.3 Education level and knowledge

Among the included studies, four identified higher educational attainment as a risk factor for adverse mental health (31, 47–49), whereas one study in India reported that populations with lower educational levels suffered more affections (50). Research conducted across seven middle-income countries in Asia highlighted risk factors for poor mental health, including being younger than 30 years, having a high educational background, being single or separated, experiencing discrimination from other countries, contacting individuals with COVID-19, and worrying about the disease (49). Another study including both high education level and single status factors reported that younger, more educated, unmarried individuals with lower household incomes were at greater risk of mental health issues (48). Educational attainment often correlates with economic status. Both highly educated individuals and those with lower socio-economic status demonstrated a higher risk of mental health problems, as did people who endorsed the view that the virus was manufactured and served specific purposes (31). However, a survey in Indian population found that individuals with lower education levels scored significantly higher on measures of depression, insomnia, and somatic symptoms, indicating a greater psychological impact of COVID-19 on their mental health and quality of life (50). Lower education levels are often associated with greater financial strain, as individuals in these groups tend to occupy lower socio-economic strata. Consequently, economically vulnerable populations facing financial instability or insecurity are at a significantly higher risk of developing mental health disorders. Meanwhile, higher education level corresponds to distinct knowledge and perceptions of the virus. Depression, stress, and anxiety were more prevalent among individuals holding master’s degrees or higher, and people with over 10 years of work experience (*p* < 0.05).

A higher level of education and a higher level of health knowledge are distinct concepts. Among front-line healthcare workers, those with more in-depth medical knowledge, compared to non-professionals, exhibited lower levels of fear regarding uncertainties surrounding the epidemic. Therefore, their psychological resilience and confidence to epidemic are higher (42). Babicki et al. (51) conducted a four-stage cross-sectional study to evaluate the prevalence of depressive and anxiety symptoms, as well as the quality of life of healthcare workers, during different phases of the COVID-19 pandemic in Poland. The findings revealed no statistically significant differences in the mean values of the BDI-II, GAD-7, and MANSA scales across the phases. However, fear associated with the disease and neighbors’ quarantines was observed to decrease.

#### 3.4.2 Population-level social determinants

##### 3.4.2.1 Prevalence of mental health issues by country and regional variations

Different economic, medical, and demographic conditions across countries can contribute to variations in mental health outcomes. The intensity of restrictions during different waves of the pandemic affected mental health in diverse ways.

Disparities in healthcare resources and variations in population demographic composition are significant factors influencing cross-national differences in mental health outcomes. Cuiyan Wang et al. (52) compared mental health outcomes in China and Iran. Revealed that Iran’s limited medical resources contributed to significantly higher levels of PTSD, anxiety, and depression across various pandemic waves compared to China, highlighting the heightened psychological burden in low-resource settings. Similarly, a study of seven middle-income countries in Asia demonstrated that limited medical resources exacerbated mental health burdens as the pandemic continued. Demographic structures also influenced mental health outcomes during different waves of the pandemic. In Iran, PTSD symptoms varied across age groups between the first and second waves. The older population experienced a notable increase in PTSD during the second wave, suggesting that different waves had a more pronounced mental health impact on older adults (36).

##### 3.4.2.2 Impact of lockdown policies

Among the involved studies, 15 studies addressed the impact of lockdown measures. In the United Arab Emirates (UAE), lockdowns, curfews, and social distancing policies significantly affected mental health, with higher levels of depression and anxiety observed (53). Malaysia implemented Movement Control Order implemented in Malaysia in March 2020 (15), and its prolonged lockdown policies contributed to increased mental health burdens (54). In South Korea, the “3 T” strategy, which involved strict quarantine measures, led to elevated levels of anxiety and depression among high-risk groups, highlighting an association between quarantining and a higher likelihood of major depressive episodes (55). Italy implemented strict lockdowns during the first wave, with gradual policy relaxations as the financial situation recovered (33). In developed countries, the duration of lockdowns and the process of transitioning out of restrictions had lasting impacts on mental health. Following lockdowns, the U. S. experienced sustained high levels of depression and anxiety, particularly among low-income individuals and households with children (45). In lower-income countries, prolonged lockdowns imposed significant psychological strain on citizens. For example, India’s strict lockdown policies resulted in notably high levels of Post Traumatic Stress Disorder (PTSD) and depression among the general population (56). In Italy and Belgium, symptoms of anxiety and depression initially increased during strict lockdowns but eased as restrictions were lifted, highlighting the psychological toll and subsequent recovery after easing measures (32, 33). However, mental health declined significantly during extended lockdown periods, particularly under the stricter conditions of the second wave, as demonstrated by a longitudinal study conducted before and during the first and second COVID-19 lockdowns in France (29).

A comparison of Israel and Italy highlighted the adverse effects of 2 months of strict lockdown on mental health and quality of life, suggesting early isolation measures had a widespread psychological impact (57). Choi et al. (58) examined the impact of lockdowns in Hong Kong SAR, China compared to mainland China, identifying significant differences. Hong Kong SAR, China’s initial lockdown and school closures had a notable impact on healthcare workers’ mental health.

The study conducted in Greece identified significant associations between pandemic awareness and mental health, with higher education and lower income groups facing more pronounced anxiety, likely influenced by lockdown policies (31). Flores-Torres et al. (59) found that not adhering to stay-at-home orders in Mexico was associated with increased mental health burdens, emphasizing the need for compliance to alleviate psychological stress.

#### 3.4.3 Social and community networks determinants

Community support, social cohesion and other informal social relationships influence mental health and resource access capacity through social capital (60). Among the included studies, four studies explored the comprehensive relations between individual and environmental factors. Lee et al. (61) investigated the socio-ecological factors associated with mental health outcomes, specifically depressive and anxiety symptoms, among individuals in South Korea during the COVID-19 pandemic to examine socio-ecological factors influencing mental health outcomes. Their findings revealed that reduced support from friends or family during the pandemic was significantly associated with increased depression (*p* = 0.0019) and anxiety (*p* = 0.0012) symptoms. Participants with increased work and home stress scored higher for depression and anxiety. Individual and interpersonal factors, such as social support and economic status, were more significant in predicting mental health outcomes than regional factors. For some vulnerable groups, low social support amplified the negative impacts on their mental health (43). Bruggeman et al. (32) examined fluctuations in mental health outcomes in response to the intensity of restrictions, focusing on vulnerable populations and identifying low social support as a key risk factor. Higher levels of both anxiety and depression were generally found among people with poor social support. From a social support perspective, being married was identified as a protective factor against depression (48, 62). A 2020 study of the German general population during the COVID-19 pandemic found that low household income and the absence of a partnership were the strongest predictors of poor mental health outcomes (30).

## 4 Discussion

The results of this systematic review underscored the profound global impact of the COVID-19 pandemic on mental health, with significant variability across countries, demographics, and policy responses. The findings reveal that the prevalence of depression and anxiety increased during the pandemic, with younger individuals (28, 36, 37, 39, 40, 42, 63), females (32, 39, 63), and socioeconomically disadvantaged groups disproportionately affected (30, 43, 45). Population-level factors, such as healthcare infrastructure (49, 52, 64, 65) and policy stringency (57, 64, 65), played crucial roles in shaping mental health outcomes. Stricter lockdowns often led to elevated psychological distress (15, 55, 57, 66), particularly in low-resource settings (52), underscoring systemic health inequalities.

Temporal patterns also emerged, highlighting how mental health outcomes fluctuated during different pandemic waves. Countries with effective early containment strategies, like China, saw improvements in mental health over time (67), while prolonged lockdowns in places like France and Slovenia correlated with worsening outcomes (13, 29). Lower-income individuals faced heightened mental health challenges (43, 45, 68), and higher educational levels correlated with distinct stress responses (31, 48, 49). Educational disparities underscored varied vulnerabilities based on knowledge and perceptions of the pandemic (31). Moreover, reduced social support significantly exacerbated depression and anxiety symptoms, emphasizing the protective role of marital status and community bonds (61, 68, 69).

The COVID-19 pandemic has profoundly affected mental health globally, with significant disparities influenced by demographic, socioeconomic, and national policy factors. These factors will act synergistically by influencing core elements such as economic livelihood security, thus forming the risk characteristics of mental health in specific groups. Some groups of the population may be more vulnerable to detrimental effects of the pandemic on mental health than others. Vulnerable populations, such as younger individuals, women, and those with lower socioeconomic status, experienced heightened levels of anxiety and depression. The work environment, and social networks showed the importance of personal factors for their mental health risk-and protection (70). Greater societal determinants of health inequality impacts their risk for disparate healthcare access and outcomes (71). Besides mental health, vulnerable populations are also at risk of poor psychological and social health underlying this definition of vulnerability is the epidemiological concept of risk. Community and associated individual characteristics are risk factors that encompass those attributes or exposures related or lead to increases in the probability of occurrence of health-related outcomes (72). These vulnerable populations will face greater risk when it comes to health outcomes beyond depression and anxiety.

The core issue of depression and anxiety stems from the lack of financial security caused by low income. The relationship between poverty and mental illness is a bidirectional causal, and the resulting concerns and uncertainties could exacerbate mental health issues (73). Factors such as age, income, living situation, and isolation can all trigger emotional issues in these specific groups, hindering their ability to maintain stable employment. As economic recessions have a context-dependent negative impact on mental health disorders (74). Young people are more likely to perceive emotional stress in sudden situations due to relatively worse psychological resilience and unstable jobs with correspondingly lower income levels. Varma et al. (75), through a global cross-sectional survey conducted in 60 countries, confirmed that younger age-groups were more vulnerable to adverse mental health outcomes. Factors such as poor sleep quality, loneliness, resilience and age emerged as mediators in the relationship between stress and mental health, highlighting these as potential targets for interventions. Kinship-loss affected population and those who are homeless are more susceptible to the impact due to their already precarious economic foundation and psychological makeup. Similarly, with underlying conditions such as emotional disorders or a history of pneumonia-related illnesses are also more likely to experience a dual impact of physiological and psychological distress. Inversely, various mental illnesses also increase the risk of multiple chronic physical diseases (76), which foreshadows the bidirectional importance and necessity of mental health practice interventions. Women, in general, due to their relatively weaker psychological endurance, tend to exhibit more pronounced fluctuations in emotional health (77), exacerbated by the additional burden of household division of labor in the role of family caregivers, intensifying the sense of crisis. The different levels of SDH often interact rather than affect independently. Young women and low-income service industry workers face compounding risks suggesting the interplay of social roles and resource scarcity.

Temporal trends and regional differences underscore the role of effective containment measures and social support in mitigating mental health challenges. Local regulations determine the state of contraction in a country or region, and corresponding lockdown policies that are either relaxed or overly strict will present different consequences, which are also related the duration. Meanwhile, these COVID-19 policies or measures are related to the economic development, and the impacts including medical, mental health and other all aspects of daily life. A full shutdown of “nonessential” activities puts market production about 25% below normal in the short run, and it also lead to employment decline (78). During the pandemic, the utilization of medical services can be reduced by nearly one third, with significant variability, and a greater reduction for less severe disease populations (79).

The interrelatedness among social groups has a significant impact on the emotional state of the population during sudden public health emergencies. Social support can stabilize emotions, and its consequences were subsumed under the general rubric of positive health states (78). The population that has been forcibly quarantined lacks interaction with the outside world, and the group of young people living alone experiences a more sense of loneliness. Reducing contact with the outside world and being exposed to more negative online news in this environment of a sudden public health emergencies can more easily lead to emotional issues. Members of the family who have lost loved ones or have family members who are ill with infections, or who face higher infection risks due to their profession, such being frontline medical staff or having family members who are, can also have a more significant impact on their emotions. However, certain studies have also demonstrated that frontline medical personnel with greater medical knowledge tend to respond to the pandemic more calmly (42) as their knowledge, cognition, and perspectives on the pandemic all play a role (31), which could also be a result of the interaction between first-level factors such as personal education level and occupational type. A certain proportion of individuals with higher educational qualifications are able to work on the frontline. These groups experience less unemployment crisis turmoil during public crisis and even gain more economic security, which responds to reduce the sense of crisis in life.

The explanatory power of SDH framework extends beyond documenting health inequalities to providing theoretically grounded guidance for designing disparity-reducing public health intervention strategies. Social-ecological model (SEM) and SDH are similar in their hierarchical classification. The social-ecological model, consisting of five levels of intervention (individual, interpersonal, organizational, community, and public policy), has been effectively in public health practice to influence behavior change and positively impact health outcomes (80). The findings emphasize the need for tailored mental health interventions that account for regional, cultural, and demographic nuances. Public health policies should prioritize vulnerable groups, such as low-income individuals and younger adults, by enhancing access to mental health services and providing targeted support programs. Ecological models of human interaction are often used to study the complex community issues that affect health inequalities (81). In epidemiology, an ecological framework is used to examine a disease as the result of disease or wellness is caused by an interaction between various factors. Structural determinants operate as root causes across ecological levels. Future interventions could adopt a structural-ecological approach, simultaneously targeting policy-level SDH levers and community-level SEM strategies, while employing ecological epidemiology to evaluate multi-factorial interactions. The review also calls attention to the importance of balancing infection control measures with strategies to mitigate psychological harm, such as promoting social support networks and providing clear communication during crises.

This study has several limitations. First, we acknowledge the complexities and lack of consensus in classifying SDH and demographic factors, including the issue of the mutual exclusivity these determinants (82). For example, we categorized housing tenure and disability status as demographic factors, acknowledging their potential classification as community-level factors considering their significant correlation socio-economic conditions. Immigration status and social capital, while categorized as community-level determinants due to their association with social policies, remain subject to disaggregation into population-level determinants. Secondly, the heterogeneity in study designs and measurement tools poses challenges for direct comparison of results. Thirdly, the reliance on self-reported measures of mental health may introduce biases, such as underreporting of symptoms. Additionally, the exclusion of non-peer-reviewed literature might have limited insights into rapidly evolving pandemic-related mental health issues.

Subgroup Considerations and Heterogeneity. Although a meta-analysis was not conducted due to the substantial methodological and contextual heterogeneity across studies, several subgroup patterns emerge from the included literature that merit highlighting. Geographically, studies from high-income countries (HICs) such as the U.S. and France frequently reported sustained psychological distress linked to prolonged lockdowns, whereas nations with early and effective containment strategies—such as China and Italy—exhibited improvements in mental health over time. Temporally, mental health outcomes fluctuated significantly across pandemic waves; for instance, Slovenia showed efficient control during the initial wave but experienced pronounced deterioration in the second wave. Methodologically, variations in measurement tools (e.g., PHQ-9, GAD-7, DASS-21) and sampling strategies (online surveys vs. population-based assessments) introduced additional layers of heterogeneity. Online studies, while necessary during lockdowns, may over represent certain demographics and underrepresent vulnerable groups with limited digital access. These subgroup differences underline the importance of contextual interpretation and caution against overgeneralization of findings. Future reviews may benefit from stratified analyses by region, income level, or pandemic phase to further elucidate disparities and contextual modifiers.

Future studies should direct greater attention toward understudied populations, such as healthcare workers and marginalized communities, to inform the development of inclusive and equitable mental health frameworks. Emphasizing the analytical role of SDH public health and disease epidemiology, further exploring the rich connotation levels and their interrelated relationships. Furthermore, more longitudinal designs should be employed to comprehensively assess the long-term psychological impacts of the pandemic. Encourage comparative studies examining the efficacy of different mental health interventions globally. Comparative analyses of policy interventions across countries could provide critical insights for managing future public health emergencies.

These findings highlight the urgent need for tailored mental health interventions and policies that address systemic inequalities and prioritize resilience-building for at-risk groups. Future research should focus on the long-term impacts of the pandemic, explore effective interventions across diverse populations, and refine strategies to foster global mental health equity.

## Data Availability

The original contributions presented in the study are included in the article/Supplementary material, further inquiries can be directed to the corresponding authors.
