# Supplementary material for: A systematic review of impacts of COVID-19 on depression and anxiety among general populations around the world
Source: Front Public Health. 2025 Oct 23;13:1659671. doi: 10.3389/fpubh.2025.1659671 (PMC12588916; doi:10.3389/fpubh.2025.1659671)
Supplement: Supplementary file 1 [file Data_Sheet_1.docx]

**Supplementary material**

**Table 1.** *Search strategy*

| **For Web of Science** |
| --- |
| TS = (coronavirus* OR COVID-19 OR covid19 OR 2019-nCoV OR 2019nCoV OR nCov-2019 OR SARS-CoV-2 OR “Severe Acute Respiratory Syndrome” OR Betacoronavirus) AND TS = (depression OR” depressive disorder “OR “depressive symptoms” OR “depressive episodes “OR depress* OR anxiet* or anxious* OR “Generalized anxiety disorder” OR GAD OR “Social anxiety”) AND TS = (“public”OR”General public” OR “General population” OR “ordinary people” OR “normal people” OR “citizens”) NOT TS = (“adolescents” OR”healthcare workers” OR”medical staff”OR”nurse”OR”meta-analysis” OR”hospital care providers” OR “patients”OR”public health”) |
| **For Embase** |
| (((('covid 19*' OR 'sars-cov-2 infection': ab,ti OR '2019-ncov infection': ab,ti OR 'coronavirus disease 2019':ab,ti OR '2019 novel coronavirus infection': ab,ti) AND ('anxiety'/exp OR anxiety OR nervousness : ab,ti OR stress s:ab,ti OR anxiousness: ab,ti OR 'anxiety disorder': ab,ti) AND ('depression, anxiety and stress scale'/exp OR 'depression, anxiety and stress scale' OR 'motional depression': ab,ti OR 'depression, emotional': ab,ti OR 'depressive symptoms': ab,ti)) AND (('general public' OR (general AND ('public'/exp OR public)) OR 'general population': ab,ti OR person: ab,ti OR people: ab,ti OR human: ab,ti OR community: ab,ti OR citizen*: ab,ti) AND ('pandemic'/exp OR pandemic OR 'global epidemic': ab,ti OR epidemic: ab,ti OR 'disease outbreaks, infectious': ab,ti))) AND 'human'/de AND 'article'/it) AND (2020:py OR 2021:py OR 2022:py OR 2023:py OR 2024:py) |
| **For PubMed** |
| (“COVID-19”[Mesh]) OR ((((((2019-nCoV Infection) OR (SARS-CoV-2 Infection)) OR (COVID-19 Virus Infection)) OR (Coronavirus Disease 2019)) OR (COVID 19 Pandemic)) OR (2019 Novel Coronavirus Infection)) AND (((((Distress) OR (Mental health problem)) OR (psychological)) OR (stress)) OR (depression)) OR (anxious) OR ((((((general public) OR (General population)) OR (public)) OR (Person)) OR (people)) OR (Community)) OR (Citizens) Filters: Abstract, Free full text AND (((((virus[Title/Abstract]) OR (viral[Title/Abstract])) OR (pandemic[Title/Abstract])) OR (epidemic[Title/Abstract])) OR (outbreak[Title/Abstract] OR infect*[Title/Abstract])) OR (coronavirus[MeSH Major Topic]) Filters: Abstract, Free full text OR ((((Mood disorder) OR (Emotion disturbances)) OR (Panic)) OR (Sadness)) OR (Emotion distress) Filters: Abstract, Free full text NOT ((((Case Reports[Publication Type]) OR (Guideline[Publication Type])) OR (News[Publication Type])) OR (Newspaper Article[Publication Type])) OR (Review[Publication Type]) Filters: Free full text AND Filters: Free full text, in the last 5 years, Humans |
| **For Scopus** |
| TITLE-ABS-KEY ( covid-19 OR *sars-cov-2 AND infection* OR 2019-ncov AND infection* OR coronavirus AND disease 2019 OR 2019 novel AND coronavirus AND infect* ) AND TITLE-ABS-KEY ( anxiet* OR nervousness OR astress OR anxiousnes ) OR TITLE-ABS-KEY ( depression OR motional AND depression OR depression, AND emotional OR depressive AND symptoms ) OR KEY ( global AND epidemic OR pandemic* OR epidemic OR disease AND outbreaks, AND infectious ) OR KEY ( general AND public OR general AND population OR person OR people OR human OR community OR citizen* ) AND Filters: PUBYEAR > 2019 AND PUBYEAR < 2025 AND ( LIMIT-TO ( DOCTYPE , “ar” ) ) |
| **For Cochrane Library** |
| MeSH descriptor: [COVID-19] explode all trees OR (COVID 19 or SARS CoV 2 Infection or 2019 nCoV Infection or Coronavirus Disease 2019 or 2019 Novel Coronavirus Infection):ti,ab,kw (Word variations have been searched) AND MeSH descriptor: [Anxiety Disorders] explode all trees OR (Anxiet* or Nervousness or stress or Anxiousness):ti,ab,kw (Word variations have been searched) AND MeSH descriptor: [Depression] explode all trees OR (motional Depression or Depression, Emotional or Depressive Symptoms):ti,ab,kw (Word variations have been searched) AND MeSH descriptor: [Epidemics] explode all trees OR (Global epidemic or pandemic* or epidemic or disease outbreaks, infectious):ti,ab,kw (Word variations have been searched) AND MeSH descriptor: [Publications] explode all trees OR (general public or General population or Person or People or human community or Citizen*):ti,ab,kw (Word variations have been searched) with Cochrane Library publication date Between Jan 2020 and Jan 2024 |
| **For CNKI** |
| (主题:民众+民众健康) OR (关:大众+普通大众(精确)) OR (篇关摘:公民+普通公民(精确)) AND (主题:流行病 +暴发性流行病 +大流行病 +全球性行病 +全球流行病) OR (篇关摘:全球流行病(精确)) AND (主题:depression+'抑郁(depression)') OR (篇关摘: 抑郁+ 抑郁状态 + 抑郁情绪 + 焦虑抑郩 +抑郁症状 +焦虑和抑郁(精确)) OR (主題:anxiety +'焦虑(anxiety)') OR (篇关摘:焦虑+焦虑和抑郁 + 焦虑程度 + 焦虑抑郁+焦虑情緒 +焦虑状态(精确)) AND (主题:COWID-19 +'covid-19'+”covid-19疫情'+'covid-19流行' +'covid-19肺炎”+'covid-19事件”) OR (篇关摘:新冠 +新冠肺炎+新冠肺炎疫情防控 +新冠疫情 +新冠肺炎疫情影晌(精确)) |

**Table 2.** *Characteristics of included studies*

| number | Source | Country | Aim | Study Type, main methods | Sample size | Measurement tool | Period of Collecting | Outcome/Key Finding |
| --- | --- | --- | --- | --- | --- | --- | --- | --- |
| 1 | Mazza, C et al. (2020) | Italy | To establish the prevalence of psychiatric symptoms and identify risk and protective factors for psychological distress in the general population. | An online survey. Multivariate ordinal logistic regression models. | 2766 | DASS-21 | 18–22 March 2020 | Female gender, negative affect, and detachment were associated with higher levels of depression, anxiety, and stress. Having an acquaintance infected was associated with increased levels of both depression and stress, whereas a history of stressful situations and medical problems was associated with higher levels of depression and anxiety |
| 2 | Rus Prelog, P et al. (2022) | Slovenia | To compare the risk for depression, anxiety, and stress during the second wave of the pandemic in Slovenia. An additional goal was to analyze the association of depression, anxiety, and stress, with the most relevant subjective factors that define the quality of life. | The data of 1,728 respondents in two samples of respondents of the second wave were analyzed using zero-inflated negative binomial regression and Mann-Whitney U-test. | 1728 | DASS-21 | Between July 2020 and January 2021 | The rise the second wave was associated with a higher risk for depression, anxiety and stress. The risk for all three was higher for younger participants |
| 3 | Maroufizadeh, S et al. (2020) | Iran | To determine prevalence of anxiety and depression and associated factors in the general population of Iran during the COVID-19 pandemic | Web-based cross-sectional study; Simple and multiple logistic regression analyses were performed to determine independent predictors of anxiety and depression. | 5328 | GAD-7, PHQ-9 | Between 17th and 29th of April 2020 | Anxiety was significantly associated with female gender, being young and middle-aged, being unemployed or a housewife, having chronic diseases, spending considerable time thinking about COVID-19, having family members, friends, and/or relatives infected with COVID-19, and death of family members, relatives or friends due to COVID-19. Same results were also found for depression |
| 4 | Orfei, M. D., et al. (2022) | Italy | Aim at investigating the mental health in the post-lockdown period in an Italian adult population and detecting demographic and psychological predictors for a worse outcome. | A cross-sectional study. | 899 | DASS-21 | September 21st and October 11th, 2020 | Lower levels of depressive, anxiety, and stress symptoms compared with data on the lockdown period; Prolonged home-working may contribute to workaholism in women and younger adults. Suggest the necessity to monitor psychological adaption over time in general and at-risk subjects |
| 5 | Lovik, A., González-Hijón, J., et al. (2023) | Sweden | to describe the mental health burden experienced in Sweden using baseline data of the | Poisson regression was fitted to assess the relative risk of demonstrating high level symptoms of depression, anxiety, and COVID-19 related distress. | 27950 | GAD-7, PHQ-9 | June 9, 2020 – June 8, 2021 | 43. 4% of participants had significant clinically relevant symptoms in at least one of the three mental health outcomes, and 7 |
| 6 | Jin, K., Huang, J., et al. (2022) | China | Explore the changes in the mental health of the public at the beginning of the pandemic and during the regular epidemic prevention and control | A longitudinal study; A chi-square test was used to compare the changes in the depression and anxiety scores at T1 and T2, and the correlation between symptoms was analyzed through Spearman's rank correlation. | 1200 | PHQ-9 | February 10, 2020 – February 18, 2020 | During the COVID-19 pandemic, part of the general population's anxiety and depression significantly reduced with time, and they rarely developed PTSD. PTSD occurrence was related to severe depression and anxiety |
| 7 | Hasannezhad Reskati, M., et al. (2023) | Iran | compare the mental health status and the COVID-19 event impact between the survivors and the general population in Mazandaran Province, Northern Iran. | A web-based cross-sectional survey; performed using convenience sampling. | 1120 | DASS-21 | From June 7 to July 27, 2020 | PTSD severity in both outpatient and hospitalized groups was significantly higher than that in the general population. Besides, the levels of anxiety and depression in the group receiving inpatient care and treatment had significantly elevated than those in the general population |
| 8 | Thomas, J., Barbato, M., et al. (2020) | the United Arab Emirates (UAE) | To identify psychosocial and specific COVID-related variables that were associated with elevated levels of depression and anxiety. A secondary aim was to assess levels of depression and anxiety, with the expectation that, relative to earlier regional surveys, symptomatology would be elevated. | An online survey and research at international level; Bivariate and multivariate associations were calculated for the main study variables. | 1039 | GAD-7, PHQ-8 | Between April 8th and April 22nd, 2020 | Levels of anxiety and depression were notably higher; Similar variables were statistically significantly associated with both depression and anxiety, most notably younger age, being female, having a history of mental health problems, self or loved ones testing positive for COVID-19, and having high levels of COVID-related anxiety and economic threat; Sections of the UAE population experienced relatively high levels of depression and anxiety symptoms during the early stages of the pandemic. Several COVID-related and psychosocial variables were associated with heightened symptomatology |
| 9 | Wu et al. (2022) | China | To investigate the related factors of subjective well-being among people in china during the regular prevention and control of COVID-19. | Network analysis; online survey then a network analysis was conducted to analyze the correlations between the sevariables | 1556 | DASS-21, SWB, BDSST | August 5 – 11, 2020 | Depression and Stress showed highest centrality (1. 29 and 1 |
| 10 | Zhu et al. (2020) | China | Investigate the public's knowledge of coronavirus disease 2019 (COVID-19) and explore their psychological status | Descriptive statistics; Pearson correlation. | 674 | DASS-21 | February 5 – 10, 2020 | The COVID-19 knowledge score and the DASS-21 total score are negatively correlated, with a specific correlation between COVID-19 knowledge recognition depression. |
| 11 | Zhou et al. (2022) | China | To investigate the public mood during Spring Festival in the coronavirus disease 2019(COVID-19) pandemic and analyze the factors that affect anxiety and depression. | Pearson correlation\logistic regression analysis\Spearman's coefficients. | 1049 | GAD-7, PHQ-9 | February 17 – 21, 2021; February 3 – 7, 2022 | During the Spring Festival in 2021 and 2022, the proportion of people with anxiety and depression in our country remained at high level. Economic income is a contributing factor to the anxiety and depression of the public |
| 12 | M. H. Flores-Torres et al. (2021) | Mexico | To describe the prevalence and correlates of depressive symptoms, generalized anxiety disorder (GAD), and perceived negative mental health impact during the SARS-Cov-2 pandemic in Mexico City and evaluate their association with adherence to stay-at-home directives. | Prevalence and Correlates study; Baseline data from a cohort study of 2,016 Mexico City government employees; multivariable. logistic regression models. | 2016 | CESD-7, GAD-7 | From June 4 to July 8, 2020 | The presence of depressive symptoms and general anxiety were associated with non-adherence to public health directives, particularly among those who might have experienced these symptoms for the ﬁrst time during the pandemic. |
| 13 | Md. Abdullah Saeed Khan (2021) | Bangladesh | Assess the prevalence and predictors of anxiety, depression and stress among young people diagnosed with COVID-19 of Bangladesh amidst the pandemic. | A cross-sectional online survey; A snowball sampling approach was used for data collection. | 974 | GAD-7, PHQ-9, PSS | May 1 – May 30, 2020 | Anxiety, depression and stress were highly prevalent among young people ( 24 years) not diagnosed with COVID-19 in Bangladesh amidst the pandemic. Unemployment is the most common underlying determinant. |
| 14 | Balvir Singh Tomar et al. (2020) | India | Assess the psychological impact of COVID-19 on mental health and quality of life of Indians | Cross-Sectional study; The multivariate logistic regression. | 2245 | DASS-21, ISI-7, PHQ-15, QoL-5 | April 28 – May 8, 2020 | Housewife had higher depression、 anxiety、insomnia 、 somatic symptoms；Front line workers had a higher psychological impact with increased scores of anxieties、stress、insomnia；Lower education level had significantly higher score in depression, insomnia , somatic symptoms. Poor physical health, social media exposure was significantly associated with heightened anxiety score. |
| 15 | S. Abuhammad et al. (2022) | Jordan | The study investigated mental health measures that include depression, stress, and anxiety, and their correlation with gender and age among Jordanians in the COVID-19 era. | A survey-based study; a quantitative, cross-sectional, and descriptive design. | 1578 | DASS-21 | April – July 2020 | The DASS scale showed significantly high scores for stress, anxiety, and depression in the groups that perceived a moderate-high probability of infection with COVID-19 ；The stress score was greater (P < 0. 001) in the men. |
| 16 | K. Souliotis et al. (2022) | Greece | To explore the knowledge and perceptions of COVID-19 in the general population in Greece, to gauge the prevalence of stress, anxiety and depression, and to examine the association between perceptions and socio-demographic variables and mental health status | A survey-based study, random, representative telephone. | 930 | DASS-21 | April 10 – 14, 2020 | COVID-19 has evoked diverse opinions in the general public, especially with respect to its similarity  to common influenza, its mode of transmission (airborne), the belief that it  is manufactured, and whether it is out of control. Respondents who believed that the coronavirus is  manufactured and those neutral towards its transmission by air, had higher  anxiety, stress and depression scores, while those who agreed that the virus  is out of control had lower stress scores. |
| 17 | Antonio P. Serafim et al. (2021) | Brazil | Investigate the prevalence of depression, anxiety, and stress symptoms, and behavioral aspects amidst the COVID-19 pandemic  in a Brazilian population." | Exploratory study. An online survey; Kolmogorov Smirnov | 3000 | DASS-21; Coping Strategies Inventory | May 22 – June 5, 2020 | These mental health problems were higher in women, people without children, students, patients with chronic diseases, and people who had contact with others diagnosed with COVID-19. ;The existence of  a group more vulnerable to situations with a high stress burden requires greater attention  regarding mental health during and after the pandemic. |
| 18 | S. Verma et al. (2020) | India | Find the prevalence rates of depression, anxiety and stress and their socio-demographic correlates among Indian population during the lockdown to contain the spread of COVID-19. | A cross-sectional survey; Binary logistic regressions \Pearson’s chi-square test\separate bivariate logistic regression analysis | 354 | DASS-21 | April 4 – 14, 2020 | Binary logistic regressions indicated employment status confidence interval and binge drinking were significantly associated with depressive symptoms; gender, employment status and binge drinking were significantly associated with anxiety symptoms; and binge drinking was significantly associated with stress symptoms. |
| 19 | Muhammed Elhadi et al. (2021) | Libya | Aim to determine the prevalence of insomnia, depressive and anxiety symptoms, and their associated factors among Libyan populations during the COVID-19 pandemic and the civil war. | An online cross-sectional survey; Logistic regression analysis\A chi-square test\a binomial logistic regression\Binomial logistic regression \Wilcoxon signed-rank test. | 10296 | GAD-7, PHQ-2, ISI | July 18 – August 23, 2020 | Depressive symptoms were significantly associated with various factors, including age, marital status, education, occupation, financial problems during the pandemic, health status, COVID-19 infection, suicide ideation, domestic violence, and lockdown compliance. Insomnia was linked to most study variables except age, education level, and occupation. Anxiety symptoms showed significant associations with age, education, occupation, financial issues, health status, COVID-19 infection, and psychosocial factors such as suicide ideation and domestic violence. |
| 20 | Leopoldo Nelson Fernandes Barbosa et al. (2021) | Brazil | To analyze the frequency of anxiety, stress and depression in Brazilians during the COVID-19 pandemic period. | A cross-sectional study; use a snow-ball sampling technique; Shapiro-Wilk normality tests \Student's t-test\Fisher's F-test. | 1775 | DASS-21 | May 15 – 22, 2020 | The symptoms of anxiety, depression and stress were identified mainly in women, single people, who did not currently work and already had some previous mental health symptom. |
| 21 | Y. Liang et al. (2020) | China | Evaluate psychological symptoms in frontline medical workers during the COVID-19 epidemic in China and to perform a comparison with the general population." | An online survey; Bonferroni’s post hoc multiple comparison test \chi-square test. | 1074 | GAD-7, PHQ-9 | February 14 – March 29, 2020 | The resilience of frontline medical staﬀ outside Hubei Province was higher than that of the general population  outside Hubei Province. |
| 22 | M. Michinaka et al. (2023) | Japan | Assess the impact of COVID-19 on mental health of the homeless in Japan by evaluating depressive and anxiety symptoms and identifying the associated factors particularly, sociodemographic variables as age, employment status and the fear and perceived risk of COVID-19 infection. | A cross sectional study; Univariate logistic regression analysis\Shapiro-Wilk test\logistic regression analysis. | 158 | GAD-7, PHQ-9, FCV-19S | April 24 – May 30, 2022 | PEH in younger age groups (18–34 years), and with joblessness, higher perceived infection risk, and higher fear of COVID-19 were more  likely to suffer from depression and anxiety. |
| 23 | D. A. AlAteeq et al. (2020) | Saudi Arabia | Explore depression and anxiety levels among healthcare providers during the COVID-19 outbreak in Saudi Arabia. | A cross-sectional study of a convenience sample; Multivariate analysis\non-parametric Mann–Whitney U tests or Kruskal–Wallis tests \ the generalized mixed linear models. | 502 | GAD-7, PHQ-9 | May 13 – September 29, 2022 | Males were significantly less predicted to have anxiety, 30–39 years age group were significantly more predicted to have depression and anxiety group, and nurses had significantly higher mean score of anxiety. |
| 24 | L. Y. Lin et al. (2021) | China | Examine the subjective sleep status and mental health of the population during the peak of the COVID-19 epidemic. | An online questionnaire; Kruskal–Wallis nonparametric analyses\ Spearman's coefficients \Hierarchical regression analysis. | 5641 | GAD-7, PHQ-9, ISI, ASDS | February 5 – May 23, 2022 | Threat degree of COVID-19 (groups) had significant correlations with insomnia, depression, anxiety, and stress (p < 0.05, p < 0.01). Age, gender, and area (Hubei province or other provinces) had significant correlations with insomnia. |
| 25 | Chun Lin et al. (2022) | China | To investigate the status and influential factors of depression, anxiety, and insomnia among people in quarantine during COVID-19. | A Cross-Sectional Study of 1,360 people in a quarantined hotel; Chi-square test \ Fisher’s exact test \ pearman correlation analysis. Multivariate analysis was performed by binary logistic regression. | 1360 | PHQ-9, GAD-7, ISI | August 2020 – November 2021 | Married was a protective factor for depression. Chronic disease was a risk factor for insomnia |
| 26 | Khademian et al. (2021) | Iran | Investigate stress, anxiety, and depression during the Corona pandemic in Iran. | An online survey; multivariate regression models\one-sample t-test and ANOVA\Pearson correlation. | 1498 | DASS-21 | April 18 – 28, 2020 | Regression model showed being female, living with a high-risk family member, health status, economic status, social capital, risk of disease, and following COVID-19 news have a relation with stress level. Education level, living with a high risk family member, health status, social capital, risk of disease, and following COVID-19 news have a relation with anxiety score. |
| 27 | Behisi et al. (2021) | Saudi Arabia | To explore the degree of association between the COVID-19 pandemic and mental health status of Saudi citizens living abroad. | A cross-sectional survey; Signiﬁcant correlations\t-test and/or Chi-square correlation test. | 662 | GAD-7, PHQ-9 | August – September 2020 | The risk of psychological symptoms was more likely experienced by females, young, single, or divorced, or those who were living alone. who lived in the UK and Ireland were more likely to develop depressive and anxiety symptoms. |
| 28 | Choi et al. (2020) | Hong Kong | To evaluate the depression and anxiety of people in Hong Kong during the COVID-19 pandemic. | A population-based study; Multiple logistic regression analysis \The Hosmer-Lemeshow test was used to assess the model ﬁt of the multiple logistic regressions. | 500 | GAD-7, PHQ-9, DSM-IV | April 24 – May 3, 2020 | Multiple logistic regression analysis found that not experiencing the SARS outbreak in 2003, being worried about being infected by COVID-19, being bothered by having not enough surgical. |
| 29 | Kim et al. (2021) | Korea | To investigate whether quarantining during the COVID-19 pandemic was associated with depression among Koreans. | A Propensity Score-Matched Analysis; logistic regression modeling approach | 919 | PHQ-9 | 2019 (Control Group); October – November 2020 (Quarantine Group) | Depression prevalence was higher in quarantined individuals than in the control group; Logistic regression analyses revealed that quarantining was associated with higher likelihoods of having major depression interval after adjusting for relevant covariates. |
| 30 | Pasha et al. (2023) | Iran | compare anxiety, stress, depression and PTSD (posttraumaticstress disorder) symptoms in diferent age groups during the Covid-19 crisis." | A cross-sectional online survey; logistic regression. | 601 | DASS-21, IES-R | December 2020 – February 2021 | The risk of PTSD in young people was higher than in the elderly, while the risk of depression, anxiety and stress did not differ significantly among the three age groups. Female gender, occupation, lower economic status, solitary life, and chronic disease were risk factors for psychological symptoms during the Covid-19 pandemic. |
| 31 | Wang et al. (2022) | China | Determine the impact of mental and psychological symptoms among population in quarantine for 2 weeks during COVID-19 pandemic. | A case-controlled study; Independent-samples T test, Chi-square test \The odds ratio (OR) of Chi-square test. | 3417 | PHQ-9, GAD-7, ISI | April 7 – June 15, 2020 | Population in quarantine showed significantly higher risks of depression, anxiety, and insomnia, when compared to the general population. Younger, more education, non-married and lower household income showed higher risks of mental health problems. |
| 32 | Amit Aharon et al. (2021) | Israel; Italy | Examine how and to what extent the severe restrictions affected the mental health and health-related quality of life of non-infected people, in a comparison between Israel and Italy. | A cross-sectional study; Linear hierarchic regression forced steps. | 1015 | PHQ-4, SF-8 | May 7 – 13, 2020 | After adjusting for socioeconomic variables, the results showed a significantly higher anxiety level and lower health-related  quality of life in the Italian participants. The anxiety and depression variables predicted lower health-related quality  of life. |
| 33 | Fiorillo et al. (2020) | Italy | Explore the effects of the lockdown on the mental health of the general population during the COVID-19  pandemic in Italy" | A survey from COMET collaborative network; multivariate regression models. | 20720 | DASS-21, GHQ-12, OCI-R, ISI, SIDAS, SASS, IES, UCLA | March – May 2020 | The depressive, anxiety and stress symptoms significantly worsened from the week April 9–15 to the week April 30 to May 4 (p < 0. 0001). |
| 34 | Gavurova et al. (2022) | Czech, Slovakia | To assess the associations of Internet addiction with depressive symptoms, anxiety symptoms, and stress in higher education students during the COVID-19 pandemic, as well as to examine these mental health problems in the context of study-related characteristics. | The Kruskal-Wallis H test\ Nonparametric tests \The Mann-Whitney U-test \the quantile regression analysis. | Czech1422；Slovakia1677 | IAT, GAD-7, PHQ-9, PSS | March – December 2020 | Internet addiction was positively associated with anxiety symptoms, depressive symptoms, and stress in all of the analyzed cases (p-value < 0. 001)；The binomial logistic regression analysis revealed that risk factors for mental health problems in Czech and Slovak students were mainly full-time form of study and living away from home during the semester. |
| 35 | Sabbaghi et al. (2022) | Iran | To investigate depression, anxiety, and stress levels of the Iranian PHEM personnel during the Covid-19 pandemic. | Descriptive cross-sectional study; one-way analysis of variance and linear regression. | 544 | DASS-21 | August – September 2021 | Depression, stress, and anxiety were more prevalent in the age group of 41-55 years, people with master’s and higher degrees, people with a history of underlying diseases, and people with over 10 years of work experience (p < 0. 05). |
| 36 | Lu et al. (2022) | French | To identify and characterize self-reported mental and physical health trajectories in the French population from pre-lockdown to the first and second COVID-19 lockdowns and to identify factors associated with health status variation patterns. | A secondary analysis; logistic or multinomial regression. | 613 | GAD-7, PHQ-9 | Before the pandemic, April – May 2020 (First Lockdown), and October – December 2020 (Second Lockdown) | Females were more likely to belong to trajectories of the most vulnerable one as regard to the symptoms of anxiety and depression, and self-perceived mental and physical health. The younger participants were also more vulnerable to anxiety symptoms and those with a clinical diagnosis or a positive COVID-19 test for the participant or relatives were more likely to belong to vulnerable trajectories for depressive symptoms and self-perceived mental health. |
| 37 | Yanmengqian Zhou et al. (2020) | United States | To examine the impact of the early COVID-19 pandemic on mental health in the U.S. and identify demographic, psychosocial, and behavioral predictors of stress, anxiety, depression, and post-traumatic growth. | A longitudinal design; Repeated measures ANOVAs\Marginal homogeneity tests\Multiple linear regressions\Standardized regression coeﬃcients. | phase1: 1021; phase2: 633；phase3: 442 | DASS-21, PTGI | Wave 1: April 20, 2020, Wave 2: May 4 – 8, 2020, Wave 3: May 18 – 22, 2020 | The early months of the U. S |
| 38 | Manfred E. Beutel et al. (2021) | Germany | Assess mental health changes, specifically depression, anxiety, and loneliness, in the German population during the COVID-19 pandemic compared to a similar pre-pandemic sample from 2018. The researchers also aimed to identify socio-demographic factors contributing to mental health risks | A cross-sectional study conducted with representative face-to-face surveys of the German general population at two time points, 2018 and 2020. The study utilized ANOVA for group comparisons and regression analyses to evaluate the statistical relevance of various socio-demographic predictors. | 2018:2516;2020: 2503 | PHQ-4 | 2018: May – July; 2020: May 2 – June 29 | Greater social strain, or less social support reported worse mental health. |
| 39 | Khubchandani, Jagdish et al. (2021) | United States | Systematically assess the prevalence of depression and anxiety in the adult U.S. population post-lockdown during the COVID-19 pandemic and identify sociodemographic characteristics associated with the mental health burden. | A cross-sectional study conducted nationwide in the U.S. via an online survey; descriptive statistics, t-tests, ANOVA, and multiple logistic regression analyses to identify predictors of depression and anxiety based on sociodemographic characteristics. | 1978 | GAD-2, PHQ-4 | Jul-20 | The prevalence of depression was 39%, anxiety was 42%, and moderate-to-severe psychological distress was 39%. Males had a higher prevalence of depression at 45%, compared to females at 32%. |
| 40 | Lee et al. (2022) | Korea | Investigate the socio-ecological factors associated with mental health outcomes, specifically depressive and anxiety symptoms, among individuals in South Korea during the COVID-19 pandemic | A cross-sectional, population-based study; Multiple linear regression models. | 1000 | PHQ-9, GAD-7 | November 5 – November 20, 2020 | Reduced support from friends or family during the pandemic was significantly associated with increased depression (p=0. 0019) and anxiety (p=0.012) symptoms; Participants with increased work and home stress scored higher for depression and anxiety (increased work stress: depression p<0.0001, anxiety p<0.0001; increased home stress: depression p<0.0001, anxiety p<0.0001); Individual and interpersonal factors, such as social support and economic status, were more significant in predicting mental health outcomes than regional factors. |
| 41 | Hubbard et al. (2021) | Scotland | Identify sociodemographic groups within the adult population in Scotland at risk of anxiety and depression during the COVID-19 pandemic. Additionally, the study aimed to determine whether loneliness, social support, threat perception, and illness representations exacerbated these sociodemographic effects​. | Cross-sectional nationally representative survey conducted via telephone in Scotland in June 2020; simple linear regression and moderation analyses. | 1006 | PHQ-4 | Jun-20 | Younger adults, women, and individuals in deprived areas exhibited poorer mental health. Moderating factors like loneliness, low social support, threat perception, and illness representations amplified the negative impacts on mental health for these groups. |
| 42 | Emily Upton, Clara De Torres et al. (2023) | Australian | Assess changes in mental health and help-seeking behaviors among young Australian adults during the COVID-19 pandemic, focusing on changes in depression and anxiety and investigating differences by gender​. | Prospective cohort study; Mixed-effect models to evaluate changes in mental health symptoms and help-seeking behaviors​. | 443 | GAD-7, PHQ-9 | May – June 2020 (Mid) | Young Increased mean PHQ-9 score from 6.0 (pre-pandemic) to 7.2 during COVID-19, with 28.5% reaching clinical significance for likely depressive disorder. Increased mean GAD-7 score from 5.0 to 5.7, with 21.2% showing clinical anxiety levels. Adults reported increased symptoms of depression and anxiety during the pandemic, but this was not accompanied by a rise in professional help-seeking, highlighting a gap in mental health service engagement for this age group. |
| 43 | Li Ping Wong et al. (2021) | Malaysia | Assess the prevalence and factors associated with the progression of mental health disorders in Malaysia during the COVID-19 pandemic, specifically focusing on depression, anxiety, and stress symptoms and their trends across various phases of lockdown measures​. | Nationwide cross-sectional survey; Temporal trend analysis of DASS-21 scores across four time periods; logistic regression to identify factors influencing mental health outcomes​. | 1163 | DASS-21 | May 12 – September 5, 2020 | A continuous rise in mental health issues, with depression escalating the most by the final phase. High-risk groups included younger adults, women, and those with perceived poor health. |
| 44 | Nadja P. et al. (2022) | Serbia | Determine whether COVID-19-related stressors, such as infection, the infection of close relatives, self-isolation, and lack of protective equipment, were associated with mental disorders, depressive symptoms, and anxiety in Serbia during the second year of the pandemic | Cross-sectional, nationally representative, face-to-face survey; Conducted in-person interviews using a multistage probabilistic household sampling in 60 municipalities. Chi-square (χ²) tests and Correlation analysis. | 1203 | GAD-7, PHQ-9, MINI | June – October 2021 | COVID-19-related stressors did not significantly increase mental disorder prevalence compared to pre-pandemic levels. However, the lack of protective equipment was weakly linked with anxiety disorders. |
| 45 | Cuiyan Wang et al. (2021) | Thailand | To assess the physical and mental health impacts of the COVID-19 pandemic across seven middle-income countries in Asia, identifying variations in mental health status and determining protective and risk factors associated with mental health outcomes. | Cross-sectional survey conducted in multiple countries; Descriptive statistics, One-Way ANOVA, and linear regression analyses. | 519 | IES-R, DASS-21 | After the pandemic outbreak in seven middle-income countries | Significant differences in IES-R and DASS-21 scores were found among the seven countries (p < 0.05). Younger age (<30), high education, single status, and perceived discrimination were risk factors for adverse mental health, while living with children, a large household, and high confidence in healthcare were protective factors. |
| 46 | Dietmar Ausserhofer et al. (2023) | Italy | Describe the progression of emotional burdens (depression, anxiety, stress) in a general population sample during the COVID-19 pandemic in 2020 and 2021. Additionally, it sought to explore the association between emotional burdens and SARS-CoV-2 infection through serological evidence | Longitudinal study; Student's t-tests and multiple regression models. | 855 | DASS-21 | June 1 – July 31, 2020; | Total DASS-21 Score: The total score decreased from 13.54 in 2020 to 11.04 in 2021, with statistical significance (p = 0.003). For participants with a confirmed SARS-CoV-2 infection in 2021 (n = 61), scores were notably higher: Total DASS-21 Score: The overall score was 16.63 for infected participants versus 9.64 for uninfected participants (p = 0.014). |
| 47 | Eugenie Sin Sing Tan, et al. (2023) | Malaysia | Assess the impact of the COVID-19 pandemic on the mental health of the general population in Malaysia, considering factors like employment status, financial instability, and income. | A cross-sectional study; Independent Samples t-Test\Chi-Square Test\Pearson Correlation Analysis. | 1246 | DASS-21, WHOQOL-BREF | January 1 – December 31, 2021 | Prolonged lockdowns and financial struggles significantly impacted mental health, with younger adults (31-50 years) experiencing higher levels of depression and anxiety. Financial insecurity was a major contributor to mental health issues. |
| 48 | Elisabet Rondung, et al. (2021) | Sweden | Assess symptoms of depression and anxiety in the Swedish population during the early stage of the COVID-19 pandemic, with a focus on identifying predictors for these mental health outcomes. | A cross-sectional study; Multiple linear regression analysis. | 1503 | GAD-7, PHQ-9 | March 26 – April 5, 2020 | The study found that younger individuals and those lacking stable income experienced higher levels of depression and anxiety. Key predictors for poor mental health included economic concerns, reduced social interaction, and decreased sleep and recovery quality. |
| 49 | Bianca T. Villalobos, et al. (2020) | USA | Document the prevalence of anxiety, depression, posttraumatic stress (PTS), and COVID-19-related fears and life impacts among Latinx adults living near the U.S.-Mexico border during the pandemic. | A cross-sectional study; Independent-Samples t-Tests\Point-Biserial and Bivariate Correlations\One-Way ANOVA. | 305 | GAD-7, PHQ-9, PCL-5, FIVE, EPII | June 24 – November 2, 2020 | Women were more affected than men, and lower-income participants (annual household income < $40,000) reported higher levels of depression (mean = 11. 95) compared to those in higher income brackets |
| 50 | Mateusz Babicki, et al. (2022) | Poland | Assess the prevalence of depressive and anxiety symptoms and healthcare workers’ quality of life during the different stages of the COVID-19 pandemic. | Four-stage cross-sectional study; The Lilliefors test\the Brownian-Forsythe test\post-hoc tests\Pearson’s chi-square test | 1243 | BDI-II, GAD-7, MANSA | April 17 – 26, 2020 | No statistically significant differences were observed in comparing the mean values of the BDI-II, GAD-7, and MANSA scales across waves. A decrease in fear due to the disease and neighbor’s quarantine was found. |
| 51 | Shanaya Rathod et al. (2023) | Arabic, Bangladesh, Canada, China, India, Pakistan, United Kingdom, Ukraine | To explore the psychological impact of the pandemic and resultant restrictions in different countries using an opportunistic sample and online questionnaire in different phases of the pandemic. | A cross-sectional online international survey. | 596；428 | GAD-7, PHQ-9, IES-R, Loneliness Scale, MSPSS | Round 1: July 1 – October 7, 2020; Round 2: January 16 – April 1, 2021 | The daily cumulative COVID-19 cases had a statistically significant effect on PHQ-9, GAD-7, and IES-R scores. These scores significantly increased in the second round of surveys with the ordinary least squares regression results. |
| 52 | Mobolaji A. Lawal et al. (2021) | Canada | Explores differences in stress, anxiety, and depression experienced by different ethnic groups during the COVID-19 pandemic. | A cross-sectional online survey; A one-way analysis of variance\Turkey’s post hoc test\Welch F test and a Games–Howell post-hoc test. | GAD-7:6944 PHQ-9:7082 | PSS-10, GAD-7, PHQ-9 | March 24 – May 4, 2020 | The burden of depression and stress were significantly higher in Indigenous populations than in both Caucasian and Asian ethnic groups. The mean difference between Indigenous and Caucasian for PHQ-9 scores was 1.79, 95% CI of 0.74 to 2.84, p < 0.01 and for PSS-10 it was 1.92, 95% CI of 0.86 to 2.98, p < 0.01). The mean difference between Indigenous and Asian for PHQ-9 scores was 1.76, 95% CI of 0.34 to 3.19, p = 0.01 and for PSS-10 it was 2.02, 95% CI of 0.63 to 3.41, p < 0.01. However, Indigenous participant burden of anxiety was only significantly higher than Asian participants’ (mean difference for GAD-7 was 1.91, 95% CI of 0.65 to 3.18, p < 0.01). Indigenous people in Alberta have higher burden of mental illnesses during the COVID-19 pandemic. |
| 53 | Gan Sing Joo, et al. (2021) | Malaysia | To examine how stress, anxiety, and depression correlate with perceived QoL among these residents. | A cross-sectional study; Pearson’s correlation test. | 180 | DASS-21, WHOQOL-BREF | July 2020 – February 2021 | A statistically significant negative correlation was found between the subscales of DASS-21 and the four domains of the QoL, with the social relationships and psychological domains showing a highly significant association (p< 0. 001). |
| 54 | Cuiyan Wang et.al. (2021) | China | To compare the severity of psychological impact, anxiety and depression between people from two develo** countries, Iran and China, and to correlate mental health parameters with variables relating to the COVID-19 pandemic | A cross-sectional, cross-country comparative study; Independent t-tests, chi-squared tests, and linear regression. | 1411 | IES-R, DASS-21 | February 28 – March 26, 2020 | Iranians reported higher levels of anxiety and depression than Chinese participants, potentially due to Iran's more severe COVID-19 situation and limited access to healthcare. Social factors, confidence in healthcare, and perceived pandemic risks also contributed to mental health disparities. |
| 55 | Helena Bruggeman et al. (2022) | Belgium | To assess the impact of the first 15 months of the COVID-19 pandemic on the level of anxiety (GAD-7 scale) and depression (PHQ-9 scale) of the Belgian adult population. | A longitudinal study; Linear mixed models. | 1838 | GAD-7, PHQ-9 | April 2020 – June 2021 | The prevalence of symptoms of anxiety and depression was higher in times of stricter policy measures; Higher levels of both anxiety and depression were generally found among women, young people, people with poor social support, extraverts, people having pre-existing psychological problems, and people who were infected/exposed to the COVID-19 virus. |
| 56 | Jin K et al. (2022) | China | To explore the changes in the mental health of the public at the beginning of the pandemic and during the regular epidemic prevention and control. | A longitudinal study; A chi-square test\ Spearman's rank correlation. | 168 | PHQ-9, SAS, PCL-5 | February 10 – 18, 2020; October 21 – December 29, 2020 | The scores of the PHQ-9 scale and the SAS scale were both positively correlated with the score of the PCL-5 scale and negatively correlated with sleep time. During the COVID-19 pandemic, part of the general population's anxiety and depression significantly reduced with time, and they rarely developed PTSD |
| 57 | Thomas J et al. (2020) | United Arab Emirates | To investigate the psychosocial factors associated with depression and anxiety in the UAE population during the COVID-19 pandemic. | Cross-sectional study; Regression analyses. | 1039 | GAD-7, PHQ-8 | April 8 – April 22, 2020 | High levels of depression and anxiety among UAE residents, especially in young adults, females, and those experiencing economic and COVID-related anxieties. |
| 58 | S. P. Singh et al. (2021) | India | Estimate the prevalence of posttraumatic stress disorder (PTSD) and depression among the general population in India due to home quarantine during the COVID-19 pandemic. | Cross-sectional descriptive study; Chi-square (χ²) Test\Pearson Correlation Coefficient. | 234 | PHQ-9, IES-R | April 25 – April 30, 2020 | Mean IES-R score was 16.37 ± 13.17, and mean PHQ-9 score was 4.77 ± 4.98. Among participants, 13.7% had clinical concern-level PTSD, 8.1% were probable PTSD cases, and 5.4% were at a level that might impact immune function. The prevalence rates of PTSD and depression were substantially higher than national averages reported prior to the pandemic. |
| 59 | C. Wang et al. (2021) | China | Compare the levels of impact of COVID-19 on mental health among people from Spain and China and correlated mental health parameters with variables relating to symptoms similar to COVID-19, COVID-19 knowledge, and precautionary measures. | A cross-sectional study; Independent samples t-test\the chi-square test\Linear regression. | 841 | DASS-21, IES-R | February 28 – March 1, 2020; April 14 – 18, 2020 | Spanish participants experienced more COVID-19-like symptoms, higher stress and depression scores, and greater use of medical services compared to Chinese participants, who reported higher IES-R scores and more discrimination abroad. Chinese participants used face masks significantly more often, while Spanish respondents' mental health was adversely affected by greater exposure to health information. |

**Table 3*.*** *Quality Assessment of the Included Studies*

| Author | Sufficient coverage of the identified sample | | | Participants sampled in appropriate way | | Simple size adequate | | describe subjects in detail | Response rate adequate | | Reliable tools | | Standard and valid measure | | Appropriate statistical analysis | | Account for all important confounding factors | | Overall |
| --- | --- | --- | --- | --- | --- | --- | --- | --- | --- | --- | --- | --- | --- | --- | --- | --- | --- | --- | --- |
| Mazza, C et al. (2020) | | N | | | N | | Y | Y | | Y | | Y | | Y | | Y | | Y | 7 (High) |
| Rus Prelog, P et al. (2022) | | Y | | | N | | Y | Y | | N | | Y | | Y | | Y | | Y | 7 (High) |
| Maroufizadeh, S et al. (2020) | | N | | | N | | Y | Y | | N | | Y | | Y | | Y | | Y | 6 (Medium) |
| Orfei, M. D., et al. (2022) | | Y | | | N | | Y | Y | | Y | | Y | | Y | | Y | | Y | 8 (High) |
| Lovik, A., et al. (2023) | | Y | | | Y | | Y | Y | | Y | | Y | | Y | | Y | | Y | 9 (High) |
| Jin, K., Huang, J., et al. (2022) | | N | | | N | | Y | Y | | N | | Y | | Y | | Y | | Y | 6 (Medium) |
| Hasannezhad Reskati et al. (2023) | | Y | | | N | | Y | Y | | N | | Y | | Y | | Y | | N | 6 (Medium) |
| Thomas, J. et al. (2020) | | | Y | | Y | | N | Y | | N | | Y | | Y | | Y | | Y | 7 (High) |
| Wu et al. (2022) | | | Y | | N | | Y | Y | | N | | Y | | Y | | Y | | Y | 7 (High) |
| Zhu et al. (2020) | | | Y | | N | | Y | Y | | N | | Y | | Y | | Y | | Y | 7 (High) |
| Zhou et al. (2022) | | | Y | | N | | Y | Y | | N | | Y | | Y | | Y | | N | 6 (Medium) |
| M. H. Flores-Torres et al. (2021) | | | Y | | N | | Y | Y | | N | | Y | | Y | | Y | | Y | 7 (High) |
| Md. Abdullah Saeed Khan (2021) | | | Y | | N | | Y | Y | | N | | Y | | Y | | Y | | Y | 7 (High) |
| Balvir Singh Tomar et al. (2020) | | | Y | | N | | Y | Y | | N | | Y | | Y | | Y | | Y | 7 (High) |
| S. Abuhammad et al. (2022) | | | Y | | N | | Y | Y | | N | | Y | | Y | | Y | | N | 6 (Medium) |
| K. Souliotis et al. (2022) | | | Y | | Y | | Y | Y | | N | | Y | | Y | | Y | | Y | 8 (High) |
| Antonio P. Serafim et al. (2021) | | | Y | | N | | Y | Y | | N | | Y | | Y | | Y | | Y | 7 (High) |
| S. Verma et al. (2020) | | | Y | | Y | | Y | Y | | Y | | Y | | Y | | Y | | N | 8 (High) |
| Muhammed Elhadi et al. (2021) | | | Y | | Y | | Y | Y | | N | | Y | | Y | | Y | | Y | 8 (High) |
| Leopoldo Nelson et al. (2021) | | | Y | | N | | Y | Y | | N | | Y | | Y | | Y | | Y | 7 (High) |
| Y. Liang et al. (2020) | | | N | | Y | | Y | Y | | N | | Y | | Y | | Y | | Y | 7 (High) |
| M. Michinaka et al. (2023) | | | Y | | N | | Y | N | | Y | | Y | | Y | | N | | N | 5 (Medium) |
| D. A. AlAteeq et al. (2020) | | | Y | | Y | | Y | Y | | Y | | Y | | Y | | Y | | N | 8 (High) |
| L. Y. Lin et al. (2021) | | | N | | Y | | Y | Y | | N | | Y | | Y | | Y | | Y | 7 (High) |
| Chun Lin et al. (2022) | | | N | | N | | Y | Y | | N | | Y | | Y | | Y | | Y | 6 (Medium) |
| Khademian et al. (2021) | | | Y | | N | | Y | Y | | N | | Y | | Y | | Y | | Y | 7 (High) |
| Behisi et al. (2021) | | | Y | | Y | | N | Y | | Y | | Y | | Y | | Y | | N | 7 (High) |
| Choi et al. (2020) | | | Y | | Y | | Y | Y | | Y | | Y | | Y | | Y | | N | 8 (High) |
| Kim et al. (2021) | | | Y | | Y | | Y | Y | | N | | Y | | Y | | Y | | N | 7 (High) |
| Pasha et al. (2023) | | | Y | | Y | | Y | Y | | N | | Y | | Y | | Y | | Y | 8 (High) |
| Wang et al. (2022) | | | N | | Y | | Y | Y | | Y | | Y | | Y | | Y | | Y | 8 (High) |
| Amit Aharon et al. (2021) | | | Y | | Y | | Y | Y | | Y | | Y | | Y | | Y | | Y | 9 (High) |
| Fiorillo et al. (2020) | | | Y | | Y | | Y | Y | | N | | Y | | Y | | Y | | Y | 8 (High) |
| Gavurova et al. (2022) | | | Y | | Y | | Y | Y | | Y | | Y | | Y | | Y | | N | 8 (High) |
| Sabbaghi et al. (2022) | | | Y | | N | | Y | Y | | Y | | Y | | Y | | Y | | Y | 8 (High) |
| Lu et al. (2022) | | | Y | | N | | Y | Y | | N | | Y | | Y | | Y | | Y | 7 (High) |
| Yanmengqian Zhou et al. (2020) | | | Y | | Y | | Y | Y | | N | | Y | | Y | | Y | | Y | 8 (High) |
| Manfred E. Beutel et al. (2021) | | | Y | | Y | | Y | Y | | Y | | Y | | Y | | Y | | Y | 9 (High) |
| Khubchandani, Jagdish et al. (2021) | | | Y | | Y | | Y | Y | | Y | | Y | | Y | | Y | | Y | 9 (High) |
| Lee et al. (2022) | | | Y | | Y | | Y | Y | | N | | Y | | Y | | Y | | Y | 8 (High) |
| Hubbard et al. (2021) | | | Y | | Y | | Y | Y | | N | | Y | | Y | | Y | | N | 7 (High) |
| Emily Upton et al. (2023) | | | Y | | Y | | N | Y | | Y | | Y | | Y | | Y | | Y | 8 (High) |
| Li Ping Wong et al. (2021) | | | Y | | Y | | N | Y | | N | | Y | | Y | | Y | | N | 6 (Medium) |
| Nadja P et al. (2022) | | | Y | | Y | | Y | Y | | N | | Y | | Y | | Y | | Y | 8 (High) |
| Cuiyan Wang et al. (2021) | | | Y | | N | | Y | Y | | N | | Y | | Y | | Y | | Y | 7 (High) |
| Dietmar Ausserhofer et al. (2023) | | | Y | | Y | | Y | N | | Y | | N | | Y | | Y | | N | 6 (Medium) |
| Eugenie Sin Sing Tan, et al. (2023) | | | Y | | N | | Y | Y | | N | | Y | | Y | | Y | | Y | 7 (High) |
| Elisabet Rondung et al. (2021) | | | Y | | N | | Y | Y | | Y | | Y | | Y | | Y | | Y | 8 (High) |
| Bianca T. Villalobos, et al. (2020) | | | Y | | Y | | N | N | | Y | | N | | Y | | Y | | N | 5 (Medium) |
| Mateusz Babicki et al. (2022) | | | N | | N | | Y | Y | | N | | Y | | Y | | Y | | Y | 6 (Medium) |
| Shanaya Rathod et al. (2023) | | | Y | | Y | | Y | Y | | N | | Y | | Y | | Y | | Y | 8 (High) |
| Mobolaji A. Lawal et al. (2021) | | | Y | | Y | | Y | Y | | Y | | Y | | Y | | Y | | N | 8 (High) |
| Gan Sing Joo et al. (2021) | | | Y | | N | | Y | Y | | N | | Y | | Y | | Y | | Y | 7 (High) |
| Cuiyan Wang et.al. (2021) | | | Y | | Y | | N | Y | | Y | | Y | | Y | | Y | | N | 7 (High) |
| Helena Bruggeman et al.(2022) | | | Y | | Y | | Y | Y | | Y | | Y | | Y | | Y | | N | 8 (High) |
| Jin K et al. (2022) | | | Y | | Y | | N | Y | | Y | | Y | | Y | | Y | | Y | 8 (High) |
| Thomas J et al. (2020) | | | Y | | N | | Y | Y | | N | | Y | | Y | | Y | | Y | 7 (High) |
| S. P. Singh et al. (2021) | | | N | | N | | N | Y | | Y | | Y | | Y | | Y | | N | 5 (Medium) |
| C. Wang et al. (2021) | | | Y | | Y | | Y | Y | | N | | Y | | Y | | Y | | N | 7 (High) |
